# Are single global warming potential impact assessments adequate for carbon footprints of agri-food systems?

**DOI:** 10.1088/1748-9326/ace204

**Published:** 2023-07-18

**Authors:** Graham A McAuliffe, John Lynch, Michelle Cain, Sarah Buckingham, Robert M Rees, Adrian L Collins, Myles Allen, Raymond Pierrehumbert, Michael R F Lee, Taro Takahashi

**Affiliations:** 1 Net Zero and Resilient Farming, Rothamsted Research, North Wyke, Okehampton, Devon EX20 2SB, United Kingdom; 2 Nature-based Solutions Initiative, Department of Biology, University of Oxford, Oxford OX1 3SZ, United Kingdom; 3 Cranfield University, Cranfield Environment Centre, Bedfordshire MK43 0AL, United Kingdom; 4 Scotland’s Rural College, West Mains Road, Edinburgh EH9 3JG, United Kingdom; 5 Department of Physics, University of Oxford, Oxford OX1 3PJ, United Kingdom; 6 Harper Adams University, Newport, Shropshire TF10 8NB, United Kingdom; 7 University of Bristol, Bristol Veterinary School, Langford, Somerset BS40 5DU, United Kingdom; 8 Agri-Food and Biosciences Institute, AFBI, Large Park, Hillsborough, Belfast, Northern Ireland BT26 6DR, United Kingdom

**Keywords:** life cycle assessment, climate change, agriculture, greenhouse gas emissions, sensitivity analysis, uncertainty

## Abstract

The vast majority of agri-food climate-based sustainability analyses use global warming potential (GWP_100_) as an impact assessment, usually in isolation; however, in recent years, discussions have criticised the ‘across-the-board’ application of GWP_100_ in Life Cycle Assessments (LCAs), particularly of food systems which generate large amounts of methane (CH_4_) and considered whether reporting additional and/or alternative metrics may be more applicable to certain circumstances or research questions (e.g. Global Temperature Change Potential (GTP)). This paper reports a largescale sensitivity analysis using a pasture-based beef production system (a high producer of CH_4_ emissions) as an exemplar to compare various climatatic impact assessments: CO_2_-equivalents using GWP_100_ and GTP_100_, and ‘CO_2_-warming-equivalents’ using ‘GWP Star’, or GWP*. The inventory for this system was compiled using data from the UK Research and Innovation National Capability, the North Wyke Farm Platform, in Devon, SW England. LCAs can have an important bearing on: (i) policymakers’ decisions; (ii) farmer management decisions; (iii) consumers’ purchasing habits; and (iv) wider perceptions of whether certain activities can be considered ‘sustainable’ or not; it is, therefore, the responsibility of LCA practitioners and scientists to ensure that subjective decisions are tested as robustly as possible through appropriate sensitivity and uncertainty analyses. We demonstrate herein that the choice of climate impact assessment has dramatic effects on interpretation, with GWP_100_ and GTP_100_ producing substantially different results due to their different treatments of CH_4_ in the context of carbon dioxide (CO_2_) equivalents. Given its dynamic nature and previously proven strong correspondence with climate models, out of the three assessments covered, GWP* provides the most complete coverage of the temporal evolution of temperature change for different greenhouse gas emissions. We extend previous discussions on the limitations of static emission metrics and encourage LCA practitioners to consider due care and attention where additional information or dynamic approaches may prove superior, scientifically speaking, particularly in cases of decision support.

## 1. Introduction

Life Cycle Assessment (LCA) is used extensively to assess the environmental impacts of various products and services. The method has been adopted widely for agri-food sector sustainability analyses (e.g. Roy *et al* 2009, de Vries and de Boer 2010) given its inherent capabilities to produce relevant decision-making information for both producers and consumers alike (McAuliffe *et al* 2020a). Biologically speaking, given the large physical size of most bovine animals (there are exceptions, e.g. Elayadeth-Meethal 2018) and their associated basal metabolic energy requirements in addition to activity needs, especially in the case of grazing cattle, they are unsurprisingly thought to be the most polluting and resource inefficient livestock globally (Poore and Nemecek 2018a). However, many global beef LCA modelling exercises are based on highly generic (i.e. regional- or national-scale) production systems using geographically and temporally averaged data, quite often emulating US-based feedlot systems which can be unrepresentative of systems elsewhere, e.g. Galyean *et al* (2011). Such LCAs, by nature, use highly uncertain emission factors and impact assessments.

LCA is increasingly informing consumer decision-making through various pathways, e.g. eco-labelling, in the context of the nutrition-environment nexus (McLaren *et al* 2021). Some of the aforementioned inherent uncertainties related to LCA and pollutant calculations can be captured to an extent through statistical methods (e.g. Monte Carlo and Taylor expansion analyses) whilst others such as nutritional *quality* and activity data, where appropriate, are more challenging to account for. Despite this, some studies occasionally draw *conclusive* recommendations to consumers and policymakers, e.g. Stylianou *et al* (2021) and in the case of nutritional metrics often adopted in LCA, Mozaffarian (2021), thus risking unrealistic or overly narrow trade-off analyses between environmental burdens and/or societal concerns being miscommunicated (globally equitable nutrition provision in the current example). This raises concerns about LCA’s role in identifying *holistically* sustainable food systems fairly and transparently. This is particularly pertinent when combined with wide-spread communication of (nutritional) LCA results on media platforms in the face of uncertainties being unavoidable and often unmeasurable due to data limitations. Regardless of whether environmentally focussed (e.g. hotspot analysis) or trade-off centric, given the importance of the impact assessment (Life Cycle Impact Assessment (LCIA)) stage within LCAs, herein we focus on agricultural greenhouse gases (GHGs) emissions and associated impact assessments, as if the numerator (e.g. GHGs) is incorrect, then all interpretations of a study will be incorrect no matter how novel or interesting the ‘functional unit’ (denominator) is. Given the overriding environmental and policy imperative to mitigate anthropogenically induced climate change, and the particular sensitivity of ruminant agriculture to climatic LCIAs as demonstrated by the range of results reported by Poore and Nemecek’s (2018a, 2018b) meta-analysis, a lowland beef production system is used as a case exemplar to test the effects of LCIA choices and ultimately, their possible implications for future agri-food LCAs.

When it comes to climate change-related impacts of agri-food products, the most common impact category considered under LCA (de Vries and de Boer 2010, McAuliffe *et al* 2020a), within the vast majority of extant peer-reviewed studies, as shown by Lynch (2019), use the 100 years global warming potential (GWP_100_), which considers the change in radiative forcing resulting from different GHG emissions integrated over a 100 years time horizon, relative to *not* producing the emission (IPCC 2013). GWP_100_ reports that biogenic methane (CH_4_), a short-lived climate pollutant, or SLCP, has a moderately powerful warming potential relative to more damaging longer lived gases (e.g. nitrous oxide; N_2_O), and has a *x*`CO_2_ equivalence (CO_2_-eq) of ∼28. The global temperature change potential (sGTP) over a 100 years time horizon (GTP_100_), another climate impact assessment indicator based on relative temperature change after the stated time horizon; however, GTP_100_ reports biogenic CH_4_ as having a CO_2_-eq of 4 (IPCC 2013). This coefficient was increased to 4.7 CO_2_-eq in more recent guidelines (IPCC 2021), (i.e. 4 herein as per 2013 IPCC 5th Assessment Report vs. 4.7 as per 2021 6th Assessment Report). N_2_O, a highly powerful GHG, also varies between GWP_100_ and GTP_100_, although less notably: 265 and 234 CO_2_-eq for GWP_100_ and GTP_100_, respectively. Note that updated values are provided in the most recent IPCC 6th Assessment Report (IPCC 2021), but in this study, we use 5th Assessment Report (IPCC 2013) coefficients for consistency with most recently published work and United Nations Framework Convention on Climate Change (UNFCCC) reporting requirements (noting that 4th Assessment Report (IPCC 2007) factors are also frequently encountered in the LCA literature).

Both CH_4_ and N_2_O differ in their radiative and chemical properties in the atmosphere, which determines how metric valuation and interpretation (e.g. weighting, grouping, and normalisation of results) changes across different impact assessment methods (and time horizons thereof). CH_4_ has an atmospheric half-life of around 12–15 years (Lynch *et al* 2020) whilst N_2_O has a half-life of around 120–150 years (Lynch *et al* 2020). With this in mind, dynamic forms of impact assessments have been suggested. For instance, consideration of how the climate impacts of various GHGs (and other impact categories indirectly affected by such gaseous emissions including acidification and eutrophication potentials) change over time (Levasseur *et al* 2010) have seen relatively little deployment in agri-food LCA studies. GWP*, a method that allows simple ‘CO_2_-warming-equivalent’ (w.e.) quantification of the dynamic impacts of SLCPs and, as highlighted in IPCC (2021), has been shown to correspond well with temperature evolution from climate models (Cain *et al* 2019), provides a novel means of reporting and appraising the climate impacts of GHGs with significantly different lifetimes.

There has recently been considerable attention bestowed upon GWP* and some deployment in agri-food case studies, demonstrated, for instance, by Barnsley *et al* (2021) who illustrated the climatic impacts of different diets over time using GWP*. However, this study, whilst useful for nutritionally focussed LCA applications, leaves an important gap in sustainability literature when it comes to clearly deploying GWP* to single commodities and agricultural production systems (particularly those associated with high CH_4_ emissions, such as beef production). The calculation method has arguably renewed interest in dynamic climate impact assessments and their potential implications on our view of climatic sustainability of various food items. The present approach intends to directly indicate warming dynamics, expressed in the form of ‘CO_2_-w.e.’ emissions: that is, we quantify CO_2_ emissions (or removals) over time that would approximate to the same temperature outcomes.

Here, we present ‘carbon footprint’ analyses of a lowland pasture-based beef production system under 90 distinct methodological scenarios and three unique LCIA (GWP_100_, GTP_100_, and GWP*). First, GWP_100_ and GTP_100_ are compared to explore the relative valuation of CH_4_ and N_2_O emissions under the two most widespread CO_2_-eq characterisation factors at present. The choice of these two characterisation factors for climate impact assessment also reflects United Nations Environment Programme and Society of Environmental Toxicology and Chemistry (UNEP-SETAC) Life Cycle Initiative guidance to report climate change impacts using both the GWP_100_ and GTP_100_, to indicate shorter- and longer-term climate impacts, respectively (Jolliet *et al* 2018). To supplement this exploration of characterisation importance (i.e. the burden value given to different climate pollutants arising from agriculture), GWP* is also applied to the 90 sensitivity analyses scenarios. This complementary assessment demonstrates a straightforward, yet informative, framework for examining GHG impacts associated with agri-food systems and provides an applied platform for other researchers to replicate in their own domains of agri-food LCA exploration.

## 2. Materials and methods

### 2.1. Goal and scope definition

The overarching goal of this paper is to demonstrate the importance of carrying out midpoint climate impact assessment sensitivity analyses, particularly when assessing *or* comparing systems which generate large amount of SLCPs, with a secondary objective being to produce a framework for other researchers to adopt. In the case of agri-food systems, this largely means CH_4_ arising from ruminant production as well as rice production, not covered herein. To validate the necessity of such an effort-intensive proposition (from a consultant’s/practitioner’s point of view), we generated a 9 × 10 full factorial virtual experiment whereby we tested the importance of CH_4_ (in terms of *Y*_m_, also known as CH_4_ conversion factor: the ratio of gross energy intake to enteric CH_4_ produced by an animal) vs. N_2_O (in terms of IPCC’s emission factor EF_1_ + EF_3PRP_, which represent amounts of N_2_O lost from the application of inorganic *and* organic fertiliser, respectively) at the cradle-to-farmgate exit system level for 90 combinations of *Y*_m_ and EF_1_ + EF_3_ of a specialised (i.e. prime) pasture-based beef production system. EF_1_ and EF_3_ were combined as they represent a single process in the farming system: fertilisation of the soil. *Y*_m_, was investigated in isolation as it has been shown to account for ∼50% or more (pending uncertainties) of total GWP_100_ carbon footprints of grassland beef systems in the UK (McAuliffe *et al* 2018). Further, these three coefficients were chosen for exploration as they have previously been shown to be the most important drivers of emissions’ uncertainty for beef production systems (Takahashi *et al* 2019).

**Figure 1.**
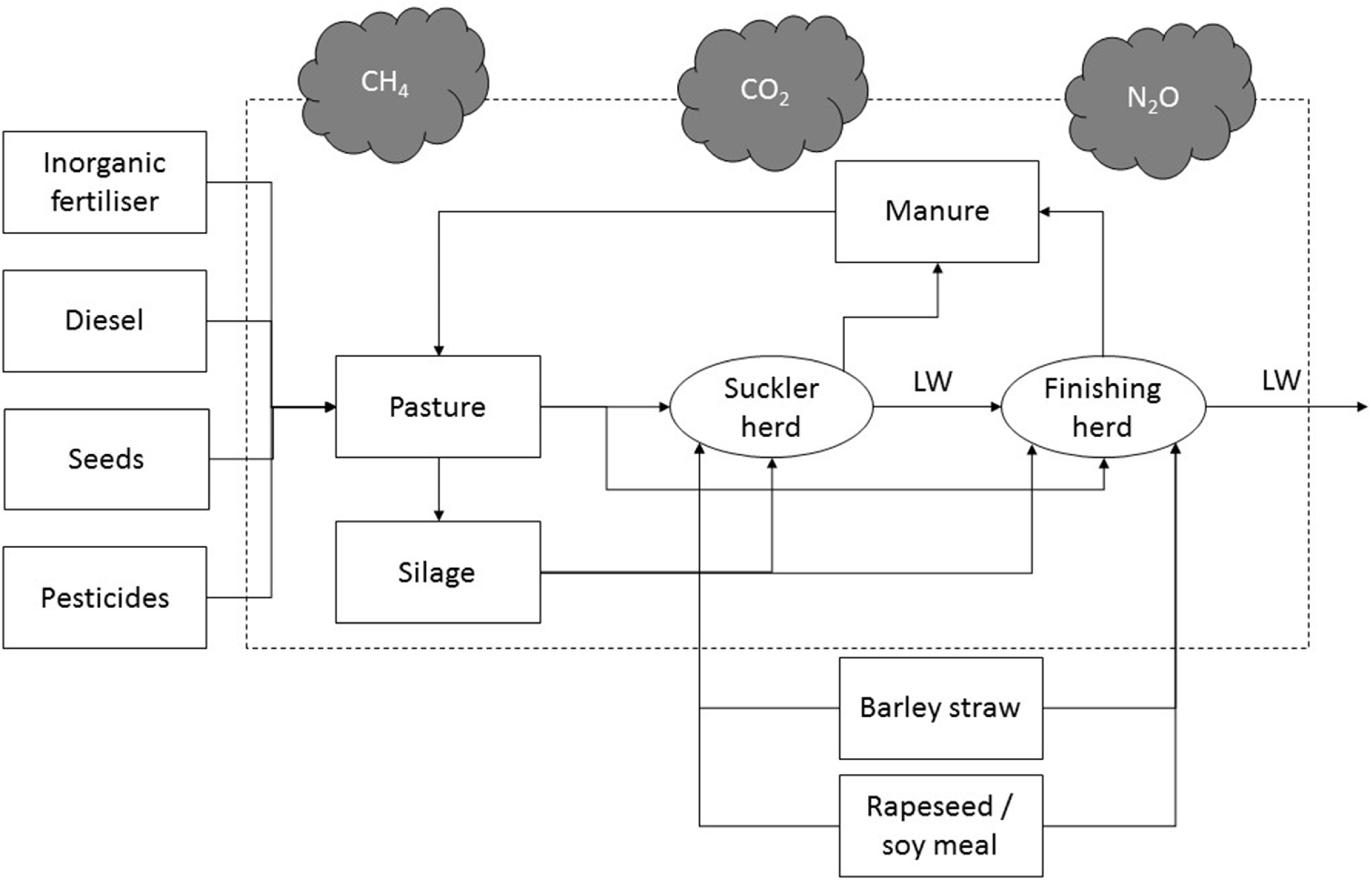
Schematic system boundary of the beef system assessed in the current study.

The functional unit was set as 1 kg liveweight (LW) exiting the finishing-cattle farmgate. In line with the functional unit, as mentioned above, the system boundary covered the extraction of raw materials (cradle) to the farmgate exit (figure 1). All material inputs and outputs, as well as losses to nature (i.e. direct and indirect GHG emissions) were covered, including those arising from the suckler herd from which the finishing animals were sourced. The only exception to the inclusion of material inputs was farm infrastructure and veterinary medicine. Both of these inputs were excluded as they have negligible effects on system wide environmental footprints of grazing beef systems as per previously published research which the current study builds upon (McAuliffe *et al* 2018). As the permanent pasture had not been ploughed for almost 100 years in some fields, with a minimum of 25 years of ley in others, soil organic carbon stocks changes were assumed to be in equilibrium as is common in grassland beef studies (de Vries *et al* 2015).

### 2.2. Life cycle inventory(LCI) analysis

All foreground data were sourced from the UK Research and Innovation National Bioscience Research Infrastructure, the North Wyke Farm Platform (NWFP). Despite having a range of long-term system trials on the NWFP, for the purposes of this study, the permanent pasture system (50°46′10″N, 3°54′05″W) was deemed sufficient to elucidate the research goal detailed in section 2.1 (figure 1). The NWFP is one of the most instrumented farms in the world for assessing the environmental performance of farming systems (Orr *et al* 2016, Takahashi *et al* 2018). The data utilised in the current study covered the 2016 permanent pasture cattle grazing system, meaning animals for finishing were born in 2015, grazed in spring and summer of 2016, and typically finished towards the end of 2016 (table 1). The finishing farm occupies approximately 21 ha, and in 2016 the beef enterprise maintained 30 Charolais × Hereford–Friesian finishing cattle sourced from an adjacent suckler herd farm which is managed in the same manner as the NWFP permanent pasture system (e.g. fertiliser rates, stocking densities etc; tables 2 and 3). All GHG emissions for 2016 were calculated according to McAuliffe *et al* (2018) using a modified IPCC (2006) Tier 2 approach, which aligns with the majority of extant grassland LCA literature.

**Table 1.** Livestock performance of finishing cattle grazing the permanent pasture system on the NWFP.

Performance	Unit	Value
Mean weaning weight	kg	332
Mean finishing weight	kg	625
Mean total growth	kg	578
Mean time spent in suckler herd	d	217
Mean time spent in finishing herd	d	408
Mean age at slaughter	d	626
Average daily gain in suckler herd	Kg d^−1^	1.31
Average daily gain in finishing herd	kg d^−1^	0.69
Average daily gain (total)	kg d^−1^	0.93

**Table 2.** Suckler herd structure and performance.

	Unit	Value
Calves	n	90.0
Heifers	n	71.5
Cows	n	127.8
Calf bodyweight^a^	kg	208
Heifer bodyweight^a^	kg	488
Cow bodyweight^a^	kg	675
Cow mortality	%	3.7
Replacement rate	%	23.6
Lifetime parity (calves per cow)	n	4.0
Culled cows^b^	kg	17 342
Weaned cattle^c^	kg	29 880
Pasture area	ha	137.1

^a^ Average bodyweight of animals during the respective life stages.

^b^ Total liveweight departing the breeding herd for slaughterhouse.

^c^ Total liveweight departing the breeding herd for finishing enterprise.

**Table 3.** Material inputs to the system and measured pasture quality for the 2016 grazing season. All values were recorded by farm staff throughout the production cycle.

Parameter	Unit	Value
Area	ha	21.61
Fertiliser area^a^	ha	21.24
FYM area^a^	ha	18.90
Pasture yield	kg DM ha^−1^	11 867
Fertiliser		
Nitrogen	kg	3354
Phosphorus	kg	257
Potassium	kg	1198
Lime	kg	3831
Rapeseed expeller	kg	1927
Straw	kg	38 728
Transport		
Rapeseed (road)	tkm	44
Straw (road)	tkm	2424
Fertiliser (road)	tkm	3698
Pasture quality		
Mean DE^b^	%	74.86
Mean CP^c^	%	23.10
Silage quality		
Mean DE^b^	%	69.40
Mean CP^c^	%	15.46

^a^ In the UK there are different field-level buffer zones designated where farmers are allowed to spread different types of fertilisers (i.e. organic or inorganic) implemented to protect environmental risks such as eutrophication which waterbodies are susceptible to.

^b^ Mean digestible energy based on multiple samples taken from pasture and silage.

^c^ Mean crude protein based on the same samples taken to analyse DE.

A large-scale sensitivity analysis capturing emission factor uncertainties was developed to assess the effect of climate impact interpretation of a typical lowland grazing beef system whilst also capturing coefficient uncertainties (i.e. *Y*_m_, EF_1_ and EF_3_) which fall within the range of latest IPCC guidelines (IPCC 2019). As the permanent pasture system also supports sheep (75 ewes plus their offspring: typically twins), on-farm impacts of sheep grazing, both positive through lamb production and associated soil fertility via excreta deposition as well as negative through GHG emissions, were separated from the model using the economic allocation-based decomposition method outlined in McAuliffe *et al* (2018). Material inputs to the system are displayed in table 3. Emissions associated with background processes, such as field activities and the production of small quantities of supplementary feeds (rapeseed expeller in the current case), were sourced from the life cycle databases *ecoinvent* (Wernet *et al* 2016) and *Agri-footprint* (Blonk *et al* 2022). Embedded CO_2_ emissions (e.g. energy consumed, fertiliser production, transportation, etc) were calculated using the aforementioned LCA databases. A 9 × 10 full factorial virtual experiment was designed to include various combinations of CH_4_ (*Y*_m_ range = 4.5%–8.5%, in steps of 0.5%, with 6.5% being the default) and N_2_O (EF_1_ range = 0.2%–2.0%, in steps of 0.2%, with 1% being default, + EF_3PRP_ range = 0.4%–4.0%, in steps of 0.4% with 2% being default) emission factors. These stepwise changes were adopted to test decision-making surrounding EFs mathematically, but it is important to reiterate that our calculations remain within IPCC’s novel recommended uncertainty ranges (i.e. 95% confidence intervals), particularly given the new system-specific seasonally- and feed-driven tailored CH_4_-yield (MY; kg CH_4_ per kg dry matter intake; DMI) calculations available from IPCC (2019).

### 2.3. LCIA

Carbon footprints were calculated for each scenario under GWP_100_ and GTP_100_ using the IPCC 5th Assessment Report (IPCC 2013) characterisation values without climate-carbon feedbacks, as these are the most commonly used and agreed upon for UNFCCC reporting. It is worth noting that IPCC’s AR6 report (IPCC 2021) has suggested changes in CO_2_-eq characterisation factors whilst, as discussed briefly in section 2.2, IPCC (2019) provides more robust calculation frameworks for estimating agricultural GHG emissions; however, the *majority* of extant LCA studies (including previous LCA work carried out on the NWFP which this study builds upon) use 5th Assessment Report characterisation factors as provided above (and which have also been highlighted for use under the Paris Agreement reporting purposes); additionally, earlier emission factors are most commonly used in existing LCA literature (e.g. EF_1_ and EF_3prp_; prp = pasture, range, and paddock; IPCC 2006). All 90 scenarios were simulated in SimaPro 8.5.2 (PRé Sustainability) using parameterisation to run multiple scenarios simultaneously. For temporal visualisations using GWP* to demonstrate ‘pulse’ (i.e. GHG emissions arising from a single production cycle) and ‘sustained’ (considering on-going, business-as-usual, production of beef over a 100 years period) emissions, only five out the 90 scenarios were presented for ease of interpretation. These were the four extremes (i.e. highest vs. lowest CH_4_ and N_2_O) and the baseline scenario which adopted default IPCC factors (i.e. *Y*_m_ = 6.5%, EF_1_ = 1%, and EF_3_ = 2%, as detailed in section 2.2). Full calculation details are available in each figure’s caption in section 3. GWP* was calculated according to Smith *et al* (2021). In addition, GWP* cumulative emissions at year 100 were calculated for each of the 90 scenarios under both pulse and sustained emissions to provide heatmaps for visual comparison with GWP_100_ and GTP_100_ as outlined in the next section.

#### 2.3.1. GWP*

GWP* is a relatively novel emission reporting approach that aims to capture the dynamic differences in shorter- and longer-lived GHGs, as outlined in the introduction. We calculate ‘CO_2_-warming-equivalent’ emissions under GWP* following the equation provided in Smith *et al* (2021):

\begin{equation*}{E^*}\left( t \right) = 128 \times {E_{{\text{C}}{{\text{H}}_{\text{4}}}}}\left( t \right) - 120 \times {E_{{\text{C}}{{\text{H}}_{\text{4}}}}}\left( {t - 20} \right).\end{equation*}

Our GWP* reported CH_4_ emissions (E*) at year *t* are calculated as the difference between CH_4_ emissions at year *t* multiplied by 128 and the CH_4_ emissions rate of 20 years previously (*t-*20) multiplied by 120. These two constants, 128 and 120, can respectively be thought of as representing the high initial impact of a CH_4_ emission (relative to CO_2_), and then an approximation of how much of the impact is automatically reversed as the CH_4_ naturally breaks down, ultimately derived from the principles of how radiative forcing responds to CO_2_ emissions (Smith *et al* 2021).

Following the recognition that the temperature response to CO_2_ shows a simple linear correlation with cumulative emissions (IPCC 2021), the ambition behind GWP* is to provide ‘CO_2_ w.e.’ of other gases that conform to this same relationship. Hence the cumulative CO_2_-w.e. in any given year following any given emission can be considered as reporting the resulting temperature increase in that year in CO_2_-eq terms. This approach provides temporal insight whilst avoiding the need for more complex time varying metrics (e.g. multiple GWP*_h_* or GTP*_h_* calculations for any and all *h* time-horizons). Further, GWP* reduces the necessity to make a subjective decision over what time-horizon should be used, given that the simple temporal approximations employed by GWP* hold reasonably well both within the 0–100 years period and beyond (as illustrated in Lynch *et al* 2020). For longer-lived gases such as N_2_O, individual emissions still act sufficiently cumulatively (over timescales up to at least a couple of centuries), meaning that the warming response is well captured without having to imagine a subsequent CO_2_-w.e. removal. The profile of N_2_O’s impacts over time decays sufficiently similarly to CO_2_ over this period; consequently, its GWP_100_ CO_2_-eq can be treated as a ‘CO_2_-warming equivalent’ quantity contributing cumulative additions to overall temperature change. When also reporting N_2_O emissions in our GWP*-based figures, these simply use the AR5 GWP_100_ CO_2_-eq value of 265 based on the aforementioned linear cumulation. See Allen *et al* (2021) for further considerations, and a comparison with a more mathematically precise approach to this equivalence.

### 2.4. Interpretation

In essence, the study as a whole is a robust sensitivity and scenario analysis combined, making it a large-scale interpretation as recommended (albeit perhaps not to this extent, which is for scientific purposes rather than product declarations etc) by ISO 14044 (2006). Here, we first compare ‘conventional’ impact assessments, GWP_100_, GTP_100_, by generating comparative heatmaps in PANDAs, a statistical dataframe-based programming module in Python.

Then, to explore the potential of GWP* in agri-food based LCAs, we employ it to provide a shorthand illustration of how climate impacts vary over time. Here, the climate change impacts were reported as a single CO_2_-w.e., a metric, as mentioned, that also generates a ‘CO_2_-eq’ quantity, but with a direct correspondence to temperature evolution over time.

Following these analyses (all based on measured data from section 2.2), two theoretical intervention scenarios were proposed and examined: (1) whether mitigating CH_4_ *or* N_2_O after 30 years is more effective at reducing the cumulative impacts of pasture-based beef production systems, and (2) whether mitigation of CH_4_ or N_2_O *first* would lead to an overall reduction in emissions over 100 years if both gases were mitigated at separate timepoints (i.e. at 30 and 50 years). In all cases of hypothetical mitigation, we imagine a transition from the highest to the lowest emission factor 95% confidence interval in our range for the relevant gas. These ranges were determined by uncertainty values provided for N_2_O in IPCC 2006 (which do not change in terms of statistical range in IPCC 2019), whilst also covering a wide range of *Y*_m_ values which overstretch the ±20% recommendation by IPCC, but is supported by unpublished research conducted at the NWFP using GreenFeed© technology to directly measure CH_4_ emissions during respiration, suggesting that the IPCC’s CH_4_ uncertainty range may be underestimated in certain soil types and microclimates). Statistical differences between GWP_100_ and GTP_100_, as well as cumulative GWP* LCIA differences at 20 years vs. 100 years, were calculated in python using a paired sample t-test.

## 3. Results

### 3.1. Scenario-based comparison of emission factor uncertainties

**Figure 2.**
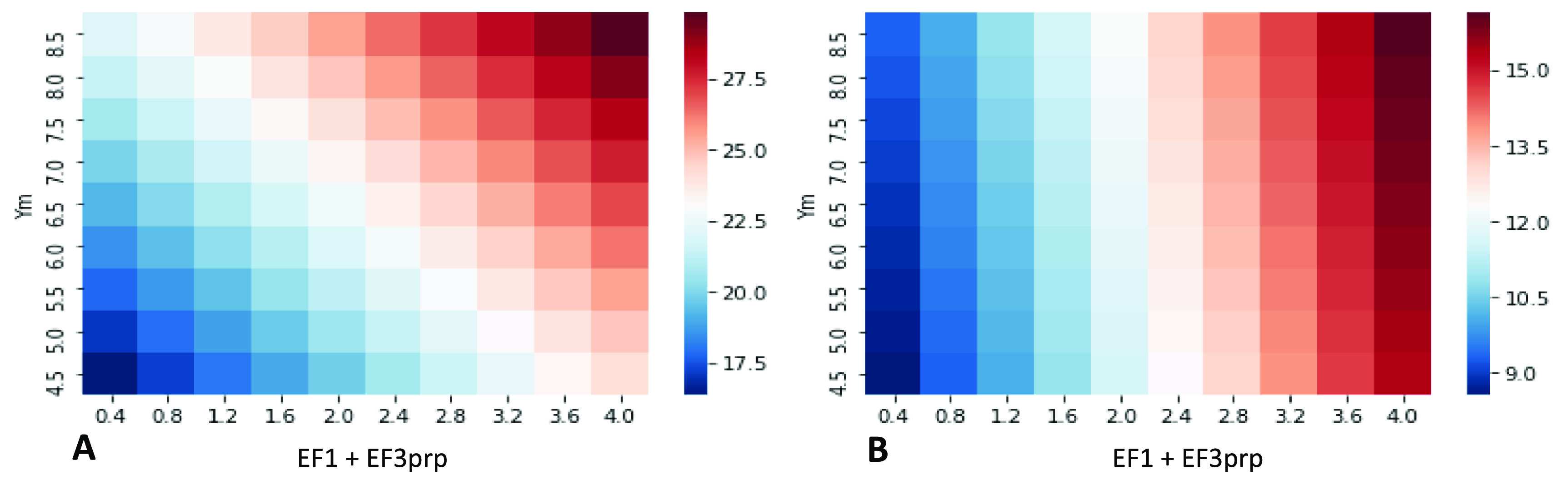
Heatmaps of all 90 scenarios described in section 2.2. Please note that the x-axis only displays EF_3prp_ emission factors (EF); however, EF_1_ ranges are also included (0.2% to 2.0% in steps of 0.2%) to represent total N applied and deposited on pasture but are not displayed for simplicity. Figure 2(A) displays impacts under GWP_100_ whilst figure 2(B) reports impacts under GTP_100_. All impacts are reported as kg CO_2_-eq/kg liveweight (LW) leaving the finishing farmgate, as calculated using the respective emission metric. prp = pasture range and paddock; *Y*_m_ = CH_4_ conversion factor; EF1 = percentage of N_2_O lost from applied nitrogen fertiliser; EF_3prp_ (PRP: pasture range and paddock) = total amount of nitrogen lost as N_2_O from grazing animal excreta deposited on grassland.

When CH_4_ occupies a significant share of emissions (as in the grass-fed lowland beef system in this study), the choice of impact assessment plays a considerable role in LCIA (figure 2). Across the 90 scenarios considered (see section 2.2), when GWP_100_ is adopted as an LCIA, the effect of enteric CH_4_ (determined in this study by *Y*_m_), a single source of GHG emissions, is comparable to the combined effect of N_2_O’s EF_1_ + EF_3_ which represent emissions arising from applied nitrogen inputs to soil (both organic and synthetic fertiliser) *and* deposited nitrogen from excreta (urine plus dung) and associated losses to nature, respectively (figure 2(A)). When CH_4_ is assigned a lower characterisation factor (i.e. CO_2_-eq), as per GTP_100_, emissions associated with N_2_O losses from fertiliser application become the dominant contributors to a lowland pasture-based beef system’s cradle-to-farmgate exit emissions intensity (figure 2(B)). It is worth noting that the choice of LCIA plays a role in the *total* emissions intensities across both methods (i.e. GWP_100_ and GTP_100_). For instance, the maximum emissions intensity reported under GWP_100_ is 29.8 kg CO_2_-eq/kg LW, whilst the maximum value under GTP_100_ is 16.1 kg CO_2_-eq/kg LW. Similarly, the average value across the 90 scenarios for GWP_100_ is 23.1 kg CO_2_-eq/kg LW with the mean for GTP_100_ being 12.3 kg CO_2_-eq/kg LW (*p* < 0.001). Lastly, it is worth noting that under GWP_100_, the total percentage contribution from CH_4_ was 39.9% whilst under GTP_100_ it was considerably lower at 9.14% under default IPCC (2013) AR5 values (i.e. *Y*_m_ = 6.5%; EF_1_ = 1%; EF_3PRP_ = 2%).

Arguments have been made for and against the use of different pulse metrics and time-horizons thereof. There is no single emission metric that can be deemed appropriate for all purposes, and any static pulse emission metric may obscure temporal detail, as discussed below. Here, we highlight that the variation in *Y*_m_, and hence CH_4_ emissions, only has a significant *proportional* impact on total emission footprints using GWP_100_ (figure 2(A)), whilst variation in EF_1_ + EF_3_, and hence N_2_O emissions, has a major *relative* impact on total footprints for both GWP_100_ and GTP_100_ (figure 2(B)) footprints, suggesting that clarifying N_2_O emissions and prioritising their mitigation may be suggested as a more universal priority, while the relative importance of CH_4_ may be more dependent upon time-horizon and/or metric of interest.

### 3.2. Temporal impacts under GWP*

**Figure 3.**
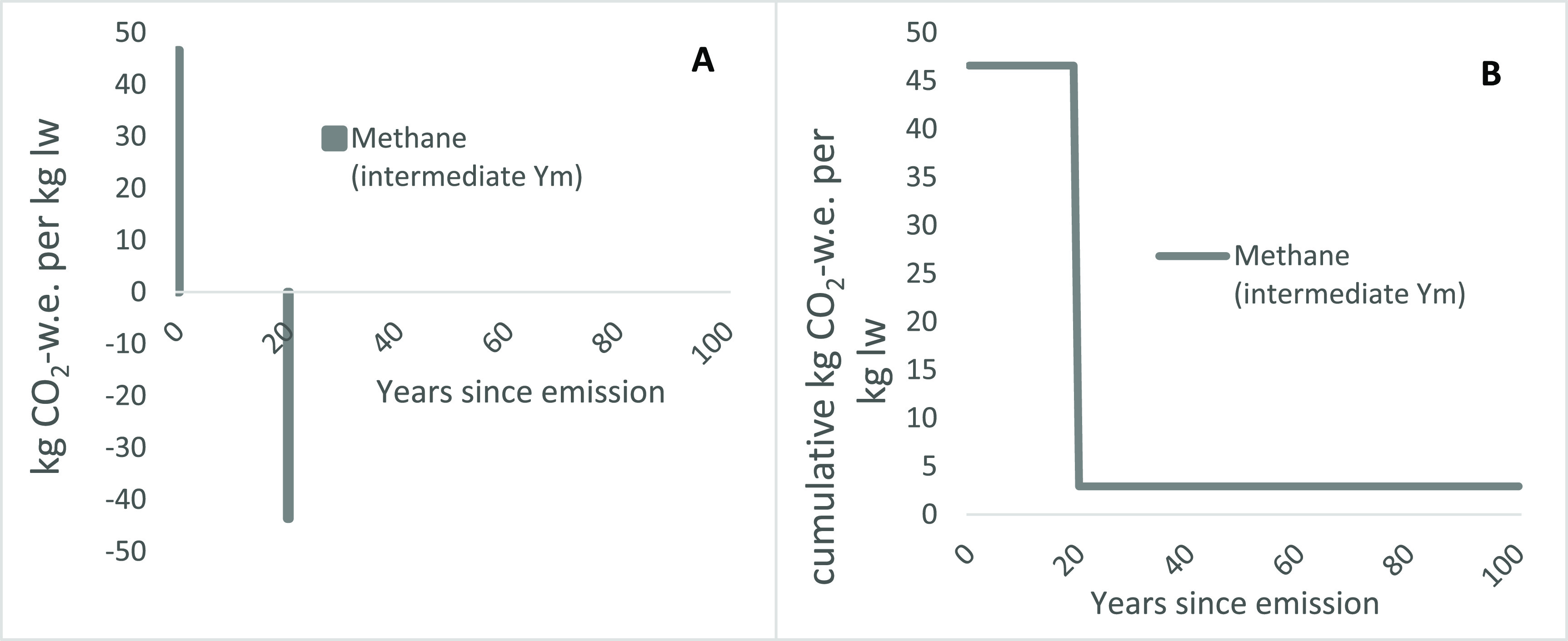
An individual pulse emission of CH_4_ for the intermediate Y_m_ footprint reported as (a) annual and (b) cumulative CO_2_-warming equivalent (CO_2_-w.e.) using GWP* for the lowland permanent pasture-based beef production system at the North Wyke Farm Platform (NWFP).

To illustrate the operation of GWP* simply, we show how a single CH_4_ emission is reported in figure 3(A), taking the CH_4_ contribution for an intermediate *Y*_m_ (6.5%) in isolation. The initial emission, in year zero, is assigned a very large CO_2_-w.e., but followed by a slightly smaller negative CO_2_-w.e. (i.e. equivalent to a CO_2_ removal resulting in a temporary ‘cooling’ effect, as expanded upon in detail by Allen *et al* 2023) in year 20. For a single pulse emission of 0.36 kg CH4, as shown in figure 2(B), this equates to 40.8 kg CO2-w.e. in the first 20 years and 2.55 kg CO2-w.e. thereafter. As noted, this simple approach approximates the initially very strong impact of a CH_4_ emissions and also their automatic reversibility due to natural atmospheric removals, both of which may be obscured via conventional treatment of CH_4_ using static emission metrics such as the GWP_100_.

It is *cumulative* emissions as reported using GWP* that have a direct correspondence with temperature change by design Cain *et al* (2019). This is because cumulative CO_2_ emissions show a simple linear relationship to temperature increases (as noted above), and therefore cumulative GWP* CO_2_-w.e. over time, as in figure 3(B), can be considered as a proxy for contributions to global temperature increase for the reported emissions at each individual year covered.

**Figure 4.**
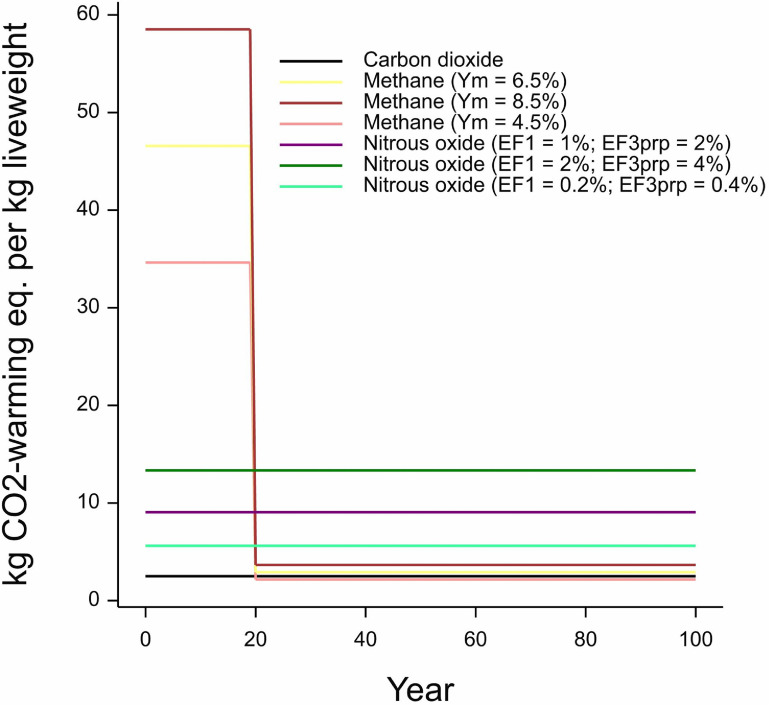
Gas-by-gas impact of a pulse emission over a 100 years time horizon calculated under GWP* for the lowland permanent pasture-based beef production system at the North Wyke Farm Platform. Y_m_ is the CH_4_ conversion factor (i.e. the proportion of gross energy lost as CH_4_ through methanogenic biohydrogenation) whilst EF_1_ represents the percentage of N_2_O lost from applied nitrogen fertiliser and EF_3prp_ (PRP: pasture range and paddock) represents the total amount of nitrogen lost as N_2_O from grazing animal excreta deposited on grassland in the current system. EF = emission factor; eq. = equivalent.

We expand on this simple illustration of the intermediate methane emission footprint to show the cumulative CO_2_-w.e. profile for all three major gases, including the intermediate value and upper and lower extremes for CH_4_ and N_2_O in figure 4, which displays a single-season ‘pulse’ emission from producing 1 kg of LW leaving the farmgate broken down by individual GHG gases as described using GWP* for 100 years after the emission. Across the first 20 years, CH_4_ is by far the dominant GHG contributing to climatic change, after which point it becomes much smaller, owing to its ∼10 years half-life, and the longer-term CO_2_-w.e. from this CH_4_ pulse is comparable to direct CO_2_ emissions from the farming system, which are typically very small (excluding embedded emissions from, e.g. ammonium nitrate production; McAuliffe *et al* 2018). From years 1 to 100, N_2_O and CO_2_ are both assumed to have the same impacts per year, with N_2_O being a considerable contributor across the entire timeframe. There is no reduction in N_2_O and CO_2_ emissions due to their atmospheric lifetimes extending beyond the 100 years time horizon covered in our study.

**Figure 5.**
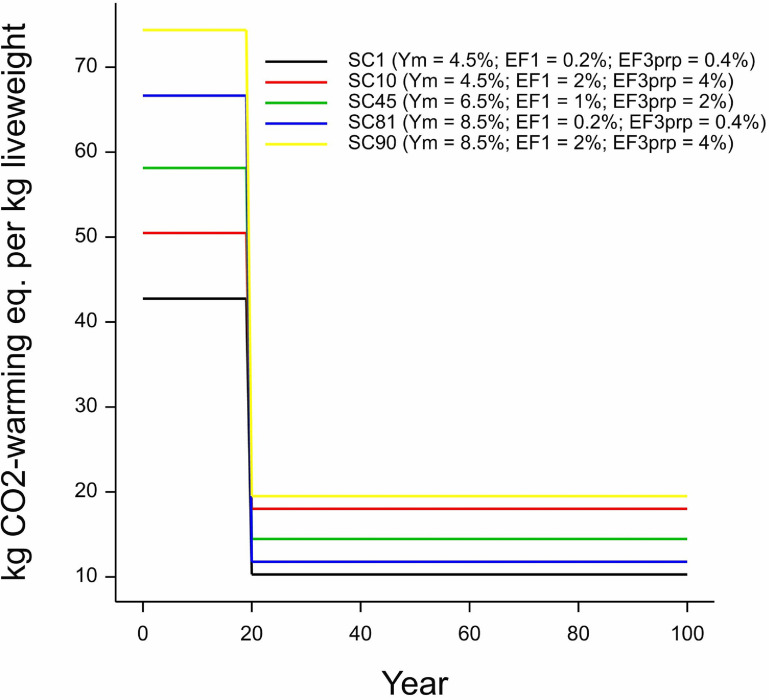
Combined greenhouse gas emissions (i.e. cradle-to-farmgate carbon footprints) for the four most extreme scenarios and the baseline scenario calculated under GWP* (section 2.3 provides more information on scenario analysis under GWP*). See figure 2 for a description of ‘*Y*_m_’, ‘EF_1_/EF_3prp_’ and ‘eq.’ SC = scenario; SC1 & SC10 are the top left and top right scenarios as per figures 2 and 7, respectively, whilst SC81 & SC90 are the bottom left and bottom right scenarios as per figures 2 and 7, respectively. SC45 reflects the central ‘cell’ in figures 2 and 7 and can be considered the ‘default’ emissions according to IPCC (2006).

Figure 5, like figure 4, displays a single pulse emission from the system under investigation. However, figure 5 represents an overall carbon footprint by summing individual gases into a single combined total (w.e./kg LW). We can see how overall warming in figure 5 follows the trends revealed from the individual gas pulse emissions displayed in figure 4, with initially significant CH_4_-dominated warming over the first 20 years, after which most of its impacts are modelled as being reversed, by a removal of ‘CO_2_-w.e.’ (Cain *et al* 2019, Lynch *et al* 2020). From here on, the overall CO_2_-w.e. declines, with N_2_O now the dominant GHG in terms of total CO_2_-w.e. emissions associated with the lowland permanent pasture-based beef system. Whilst exploring a single pulse emission is interesting from the perspective of examining the behaviour of individual gases, and is the basis of most standard emission metrics, it does not tell us very much about sustained production which is expected to be required of most agri-food systems to feed an ever-growing population (Gerber *et al* 2013); nor does it provide a more realistic scenario of time-series emissions.

**Figure 6.**
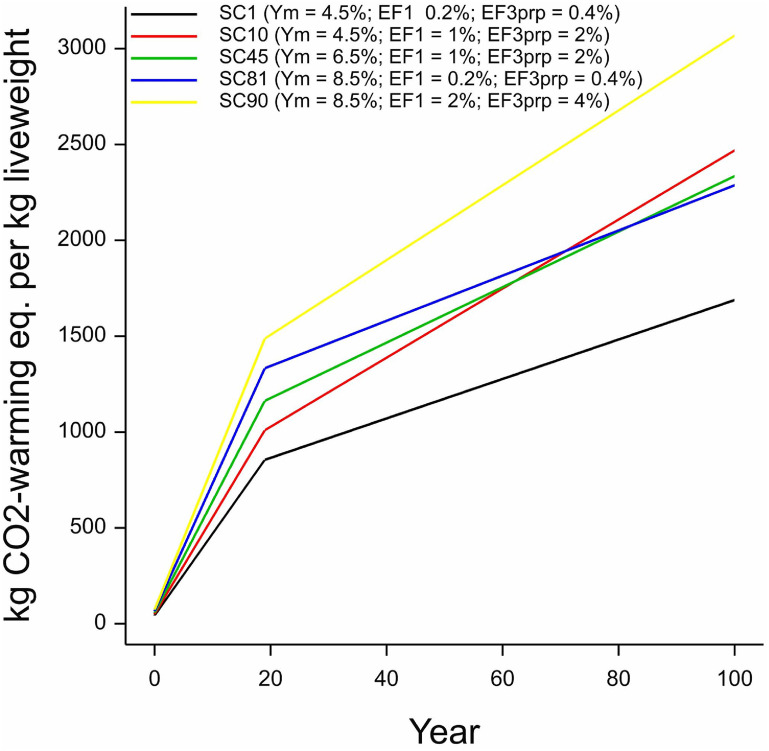
Cumulative emissions calculated for the five scenarios addressed under GWP* (section 2.3). This analysis assumes a steady-state level of beef production emissions efficiency under each scenario. SC = scenario; SC1 & SC10 are the top left and top right scenarios as per figures 2 and 7, respectively, whilst SC81 & SC90 are the bottom left and bottom right scenarios as per figures 2 and 7, respectively. SC45 reflects the central ‘cell’ in figures 2 and 7 and can be considered the ‘default’ emissions according to IPCC (2006).

To understand how ongoing emissions would be reported under GWP* as an LCIA, we modelled beef production under constant (steady state) production (figure 6). As shown in figures 4 and 5, the most rapid increase in reported emissions intensity occurs during the first 20 years when the impact of establishing the new CH_4_ source is highlighted as leading to a significant rate of warming and is consequently the key driver of the overall GWP* footprint. From year 20 onwards, the trajectory of reported emissions reduces and, future increases are driven predominately by N_2_O and CO_2_. The sustained emissions (figure 6) also show a cross-over between the blue (high *Y*_m_ & low EF_1_ + EF_3_) and red (low *Y*_m_ & high EF_1_ + EF_3_) lines at around year 60 indicating the total impacts of the long-lived, accumulating N_2_O emissions starts to exceed those of SLCPs, i.e. non-accumulating CH_4_ emissions. This dynamic occurs despite emissions of both gases continuing at the same rates each year: hence the temporal change in relative significance would not be revealed through static metrics such as the GWP_100_ or GTP_100_ that are defined by the impacts across or at 100 years only.

**Figure 7.**
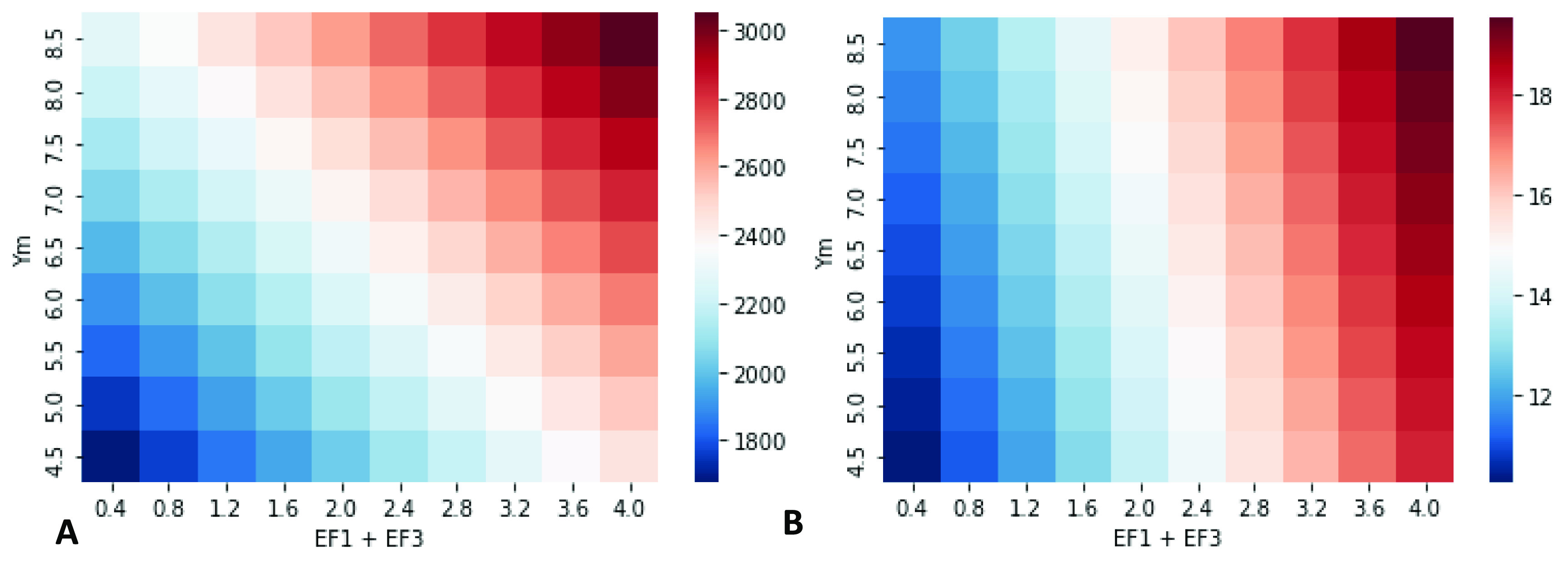
Heatmaps of all 90 scenarios, as in figure 3, but with two sets of GWP*-reported emissions. Figure 8(A) shows cumulative GWP* CO_2_-w.e. in year 100, following sustained emissions (as determined by each square’s *Y*_m_ and EF_1_ + EF_3_ combination) at the same rate every year from years 0 to 100. Figure 8(B) shows cumulative GWP* CO_2_-w.e. in year 100, following a pulse emission (as determined by each square’s *Y*_m_ and EF_1_ + EF_3_ combination) in year 0, and no subsequent emissions. All impacts are reported per kg liveweight (LW) leaving the finishing farmgate in the respective scalar legends.

**Figure 8.**
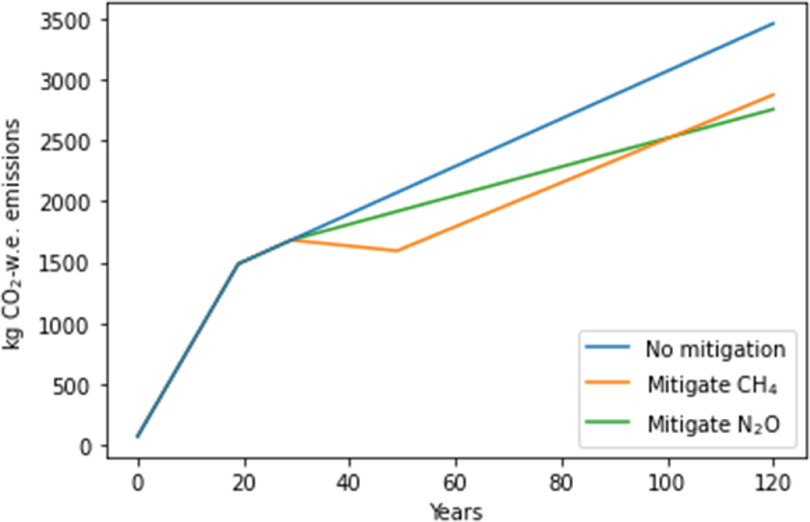
Hypothetical virtual experiment to determine which GHG (i.e. CH_4_ or N_2_O) would be more appropriate to mitigate at year 30 over a 100 years time horizon. w.e. = warming equivalent.

The heatmaps in figure 7 reveal some of the links between GWP* and pulse-emission metrics aided by generating results for 100 years from figures 4 and 5 across the full range of all virtual scenarios. Figure 7(B) shows the cumulative GWP* CO_2_-w.e. reported for all virtual scenarios occurring in year 100 following a pulse emission of the GHG footprint: i.e. the same CO_2_-w.e. in figure 5 at year 100; the five values shown in figure 5 correspond to the four corners and central value of the heatmap in figure 7(B). Figure 8(B) is very similar to the GTP_100_ footprint in figure 3(B), as expected, as they convey the same information: CO_2_-eq temperature change contribution at a certain number of years following a set of pulse emissions.

As shown in figure 6, GWP* provides a shorthand approximation of how these change over time, rather than having to calculate a full temporal evolution of GTP for every year *x.* Figure 7(A) shows the GWP* CO_2_-w.e. from all virtual scenarios occurring in year 100 following sustained emissions of the GHG footprint: i.e. the same CO_2_-w.e. in figure 7 at year 100. Figure 7(A) shows a similar pattern to the GWP_100_ footprints in figure 2(A) but increased by around two orders of magnitude. This broad increase can be intuited straightforwardly as expressing CO_2_-eq impacts of 100 years’ worth of recurrent emissions *vs* a single annual pulse. A similar relative valuation across different scenarios is also as expected, recognising some further connections between different emission metrics: as employed here, GWP* is essentially providing a shorthand equivalent to the 100 years sustained sGTP—the relative contribution to temperature change resulting from emissions sustained at the same rate for the defined number of years, which results in a similar ratio to the GWP (as observed in Azar and Johannson 2012), and thus we see a similar spread in results when the same time-horizon (100 years) is used for both. The utility of GWP* is to provide a simple and intuitive way to explore time-varying temperature impacts, with a greater universality than GTP*_h_*, sGTP*_h_* or GWP*_h_* for a given time-horizon. Moreover, GWP* is applicable for any range of emission scenarios, not just the simple pulses and sustained emissions as illustrated here.

### 3.3. Emission reduction scenarios

We expand our analysis and highlight the potential for GWP* to reveal temporal dynamics and trade-offs by exploring potential mitigation strategies. Figure 8 shows trajectories of CO_2_-w.e./kg LW if hypothetical emission reduction interventions were introduced at year 30 for CH_4_ *or* N_2_O. In both cases our imagined mitigations involved moving from the upper to the lower ends of the emissions uncertainty range, to indicate a realistic range in mitigation potentials (i.e. the CH_4_ mitigation moves from high *Y*_m_ to Low *Y*_m_, while the N_2_O mitigation moves from high EF_1_ + EF_3_ to low EF_1_ + EF_3_). When the CH_4_ mitigation scenario is adopted (orange line), compared to no mitigation (blue), we observe a large initial drop in CO_2_-w.e., as due to CH_4_’s short-lived nature, most warming it causes is rapidly reversed (i.e. there is a temporary ‘cooling’) once emissions cease. Meanwhile, for N_2_O mitigation (green line), reducing emissions slows the rate of warming, but due to its long-atmospheric lifetime, we do not achieve a reversal of the warming caused by past emissions (at least within the time-horizon explored here, and the simplifications employed by this CO_2_-warming-equivalent approach). Nevertheless, if production continued as-is following either single intervention, thus leaving emissions of the other gas to be continued unabated, eventually—from around year 100 onwards—we would be better off having mitigated N_2_O instead of CH_4_, with the accumulating benefits of reducing the long-lived, cumulative gas outweighing the initial advantage shown for reducing CH_4_. However, it is worth reiterating that this is a virtual experiment and in reality, multiple mitigation strategies will, or should, be deployed together and importantly, those would need to consider potential co-benefits and trade-offs for other environmental consequences of agri-food systems, beyond GHG emissions alone.

**Figure 9.**
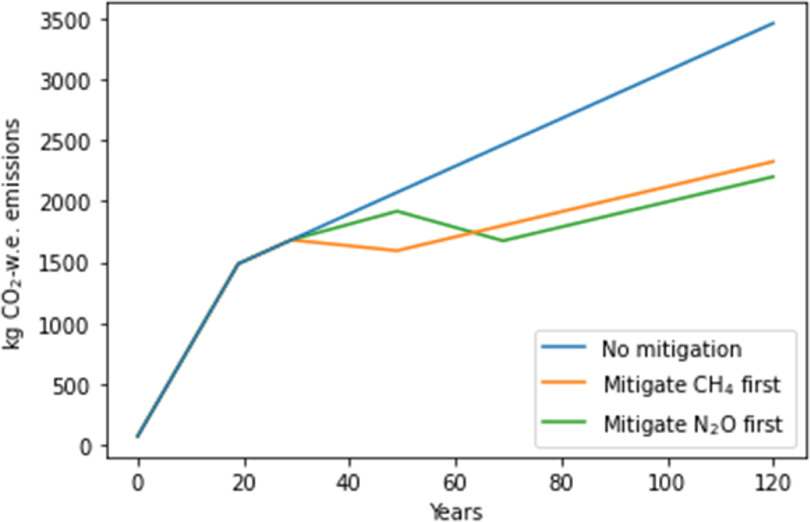
Virtual experiment to investigate whether it is more prudent to hypothetically mitigate CH_4_ or N_2_O first. Hypothetical interventions are introduced at years 30 and 50. w.e. = warming equivalent.

Another hypothetical experiment explores interventions carried out at years 30 *and* 50, with either CH_4_ or N_2_O being reduced first, followed by the other gas 20 years later (figure 9). Again, we observe the greater initial temperature decrease when acting upon CH_4_ first (orange), but if we abate N_2_O first (green), we see more greatly reduced warning for all periods beyond this initial short-term window, it was better to prevent the accumulation of N_2_O emissions first, rather than prioritising emission reductions for non-accumulating CH_4_, as most of the temperature-reversal benefits of reducing CH_4_ emissions are still achieved even if CH_4_ emission reductions are delayed. The wider context of these or other potential mitigation decisions should be interpreted with caution however, since we do not consider the broader consequential impacts that these hypothetical mitigations may be associated with in real life; for example, land use to facilitate carbon uptake such as via agroforestry/woodland expansion or other land cover transitions resulting from agricultural system change are not accounted for in the current study. Nevertheless, the results presented in figure 9 are consistent with observations made in Lynch *et al* (2020) which compared CH_4_ with CO_2_ rather than N_2_O.

## 4. Discussion

### 4.1. Implications for GHG assessments of CH_4_-intensive production systems

As shown above, this subjective decision (i.e. LCIA choice) has a considerable effect on the interpretation of a given study and demonstrates the necessity for LCIA sensitivity analyses. The simplistic use of single, static CO_2_-eq valuations of CH_4_ inherent in these assessments suggests that environmental scientists and systems analysts need to work diligently to be better-able to account for and report the complexities of individual gases in the atmosphere. Given the influence of environmental issues to consumer decision-making, the importance of these calculations cannot be underestimated. Recent high-profile studies (e.g. Poore and Nemecek 2018a) have taken a marked first step in standardising global LCAs (Poore and Nemecek 2018b); however, the true climate response is not sufficiently tractable due to aggregation of different gases with simple, static metrics, and we argue more could be done to address some of these complexities by the LCA community (e.g. through emission factor and LCIA sensitivity analyses as described above). GWP*, as outlined with simple examples in section 3.2, further provides a simple but effective tool to break the impact of each GHG down across any timeframe by providing a simple ‘warming-equivalent’ approach.

It should also be noted that these GWP* examples essentially show the warming that would result from our hypothetical emission scenarios being introduced with no prior emissions. In reality, any emitters’ contribution to global warming, and overall global temperature change, are also a function of past emissions. For long-lived gases this is broadly a simple function of cumulative emissions that result in long-term warming, but for SLCPs, the warming behaviour is more dynamic, as past emissions are continuously removed, and climate impacts depend more on the current flow of emissions. The year-on-year temperature change resulting from a certain quantity of CH_4_ differs markedly depending on whether this is a newly established source, or an existing flow of emissions being maintained, for which the pronounced initial temperature increase is already experienced.

This behaviour is revealed through GWP*: CH_4_ emissions, even if emitted at the same annual rate (as in figure 6, for example), are reported as ‘CO_2_ warming-equivalent’ emissions that change over time so they would result in approximately the same dynamic temperature changes as the methane emissions themselves would cause. However, as individual methane emissions are reported as different ‘CO_2_ warming-equivalents’ according to temporal context, Rogelj and Schleussner (2019) argue GWP* may lead to ‘unintentional unfairness’ due to the valuation of methane in a given year being contingent on past emission rates. Cain *et al* (2021) and Allen *et al* (2021) counter that this context-dependence is necessary to facilitate true ‘warming-equivalent’ comparisons and hence can itself reveal relevant equity concerns, and that a more transparent way of overcoming concerns about equitable entitlements of methane emissions themselves is simply to report and compare methane emissions directly, which can be done independently of any CO_2_-eq metric. Alternatively, the framing presented here is not only applicable for considering the establishment of a new emission source (e.g. a new farm resulting from land use change) in year 0, but also represents the ‘marginal’ future warming that could potentially be avoided from contemporary and future emissions (or reductions thereof), following the terminology employed by Reisinger *et al* (2021). This ‘marginal’ approach (employing GWP* with a zero-emission baseline) shows future impacts independently of any past emissions and warming they may have caused, and thus methane emissions from different emitters would always be reported in the same way independently of past context.

These dynamics have wider implications on how overall warming contribution is perceived and what might be necessary for different emitters to achieve climate-related ‘sustainability’, or to meet certain temperature targets (Lynch *et al* 2021). On the other hand, it has also been argued that, despite the advantages of GWP* in more accurately reflecting the temperature impacts of CH_4_ emission pathways over time, the metric would not provide a full alignment between the Paris Agreement’s mitigation mechanisms (a metric defined as a ‘balance between anthropogenic emissions […] and removals’) and long-term temperature goals (Schleussner *et al* 2019). Allen *et al* (2022a, 2022b) explore some of the context for different ‘net-zero’ definitions and challenges in trying to align conventional emission metrics with temperature-based goals.

Full discussion of these points is beyond the scope of this paper, however, as we (a) focus on providing a stepwise approach to calculating GWP* in agricultural circumstances, and more scientifically speaking, (b) demonstrate more applied comparison of the key gas dynamics and simple cases of how they are reported in emission metrics; yet, we highlight some of the broader considerations that become apparent following more detailed interrogation of how different emissions operate than would be possible using static pulse-emission metrics. A CO_2_-w.e. approach, such as GWP*, provides a straightforward means of exploring these issues and could be used to compare different framings, but implications and most appropriate context are still questions that cannot be resolved by metric selection in and of itself (in other words, not one metric suits all research questions, hence our recommendation that LCIAs should be rigorously tested for sensitivity and reported accordingly).

### 4.2. Recommendations based on current findings: are single GWP metrics sufficient?

As above (section 4.1), we argue that using a single static impact assessment (LCIA in the context of LCA) is insufficient to elucidate the complexities of how agri-food systems contribute to climate change. Instead, consideration should be given to representing these complexities by providing a *range* of metrics. This may include reporting individual GHG emissions independently of each other for a reader’s ease of interpretation thereby enabling them to undertake their own climate impact interrogations (Lynch 2019) which adequately demonstrate the trade-offs associated with different approaches. For instance, long versus short-term impacts and/or potential benefits of mitigation interventions such as novel ‘sustainable’ fertilisers (e.g. biochar; Kammann *et al* 2018) and methane inhibitors including 3NOP (e.g. Lopes *et al* 2016). Given the importance of farming to international food security, there is no doubt that the sector needs to optimise productivity (McAuliffe *et al* 2018, Lee *et al* 2021) whilst minimising energy-intensive inputs such as inorganic fertiliser (McAuliffe *et al* 2020b). This importance is demonstrated by the range of GHG impact efficiencies across the globe under various production systems, some of which can be quite inefficient (Poore and Nemecek 2018a, 2018b). However, the evidence presented above suggests that much of the information currently being communicated to stakeholders and laypeople alike may provide an incomplete or, potentially even misleading, representation of the impact of agriculture towards climate change (section 4.3).

As a result, the first major recommendation resulting from this study is that LCA practitioners, national inventory compilers, and other sustainability scientists calculating environmental burdens of agri-food systems need to test the robustness of assumptions by adopting multiple sensitivity analyses (e.g. in terms of LCIA: GWP_100_, GTP_100_, and GWP*; figures 2 and 7, respectively), whilst also reporting GHG emissions individually (Lynch 2019). At a minimum, CO_2_-eq emissions must be reported separately for long- and short-lived GHGs, as it is widely acknowledged that without this disaggregation it is not possible to infer temperature outcomes, and hence stymies comprehensive communication surrounding the implications of any emissions mitigation measures for global climate targets (Allen *et al* 2022b).

GWP* offers a complementary approach to calculate GHG emissions over time; however, further standardised applications of GWP* are required, and its *widespread* uptake is unlikely in the short term. In the meantime, in addition to reporting GWP_100_ and GTP_100_ simultaneously, scientists including inventory compilers should consider calculating emissions over various time-horizons (e.g. GWP_20_ and GWP_500_). Whilst this undoubtedly adds an additional layer of complexity to the interpretation of such studies, by focussing solely on GWP over 100 years, the manner in which the relative impacts of CH_4_ vs CO_2_ and N_2_O change over time is currently unaccounted for. One aspect of this is that using a 100 years horizon alone also fails to reveal the full significance of the short-term gains in terms of reduced planetary warming of targeted mitigation of CH_4_ as recommended in the final *comunicae* of the COP26 meeting in Glasgow (UN 2021).

### 4.3. Conclusions and scope for further research

In recent decades, LCA, and in particular related LCIs, have gained scientific robustness and sophistication (e.g. via increased awareness and consideration of uncertainty throughout supply chains); additionally, the potential for wider system boundaries (i.e. broader supply chain coverage) is made possible through the evolution of existing LCA databases such as *ecoinvent* and development of newer, agriculture-specific databases (e.g. *Agri-footprint* and *Agribalyse*). Further, as discussed earlier, there is a growing consensus that sustainability assessments of agri-food products should account for wider components of global burdens, not least societal issues (e.g. Costa *et al* 2020, McAuliffe *et al* 2020a). Stepping beyond the boundaries of the current study for a moment, considerable advancements have been made when tackling complex issues such as allocation in LCA-based agri-food systems which produce multiple products (e.g. milk and meat in the case of dairy production; Thoma *et al* 2013, Rice *et al* 2017, March *et al* 2021). Further, whilst in its infancy, nutritional- and health-based LCA (known as nLCA) has the potential to account for human health. This is often achieved *indirectly* through assessing the variability of nutritional *quality* of food commodities (Sonesson *et al* 2017, McAuliffe *et al* 2023), in addition to *direct* human health impacts arising from environmental burdens simultaneously via trade-off assessments (e.g. Stylianou *et al* 2016, Sonesson *et al* 2019).

Bearing the above advances in mind and given that, according to the FAO, ∼38% of global terrestrial land excluding icecaps is dedicated to agricultural activities, there is an urgent need to generate up-to-date environmental, economic, and social sustainability assessments of the UK’s major agricultural commodities, particularly as around 70% of land in the UK is used for agriculture (CIEL 2020). The methodological case study provided herein demonstrates one such advancement in agri-food environmental sustainability by providing better insights into GHG emissions’ behaviour in the atmosphere. Perhaps of equal importance, we encourage LCA scientists to test their subjective choices more rigorously including impact assessments and reporting LCIA sensitivity analyses in future studies, particularly when SLCPs are significant system-wide GHGs; for instance, the ultimate potential for ‘sustainable’ ruminant livestock systems and rice paddies may be viewed differently through the adoption of GWP* during LCIA when compared to GWP_100_ or indeed GTP_100_, thus providing ample scope for applied GWP*-based LCAs on global food items (i.e. national staple food commodities and emerging alternatives such as plant-based proteins).

The importance of testing LCA modelling subjectivity (which, for clarity, cannot be avoided but can be assessed and reported transparently) through robust sensitivity analyses *and* simulation-based statistics such as Monte Carlo cannot be underestimated as, demonstrated presently, these choices have drastic effects on LCA interpretation and subsequent communication (e.g. through ‘eco-labelling’), which often misleads consumers (Steenis *et al* 2017) and policymakers (Cederberg *et al* 2011) due to modelling inconsistencies and a focus on single impact categories, primarily climate-related impacts (Nemecek *et al* 2016). Finally, a lack of attention to broader sustainability issues (Costa *et al* 2019) such as those discussed above in the current section and covered in detail by McLaren *et al* (2021), e.g. human health, agricultural resilience, nutritional complexities, and ultimately, and global food security requires LCA practitioners to be aware of, and take better care in, communicating limitations of their studies, which are often overlooked or unreported.

## Data Availability

All data that support the findings of this study are included within the article (and any supplementary files). Underlying data used in this study to develop our inventory analysis are publicly available via the North Wyke Farm Platform Data Portal: https://nwfp.rothamsted.ac.uk/.
